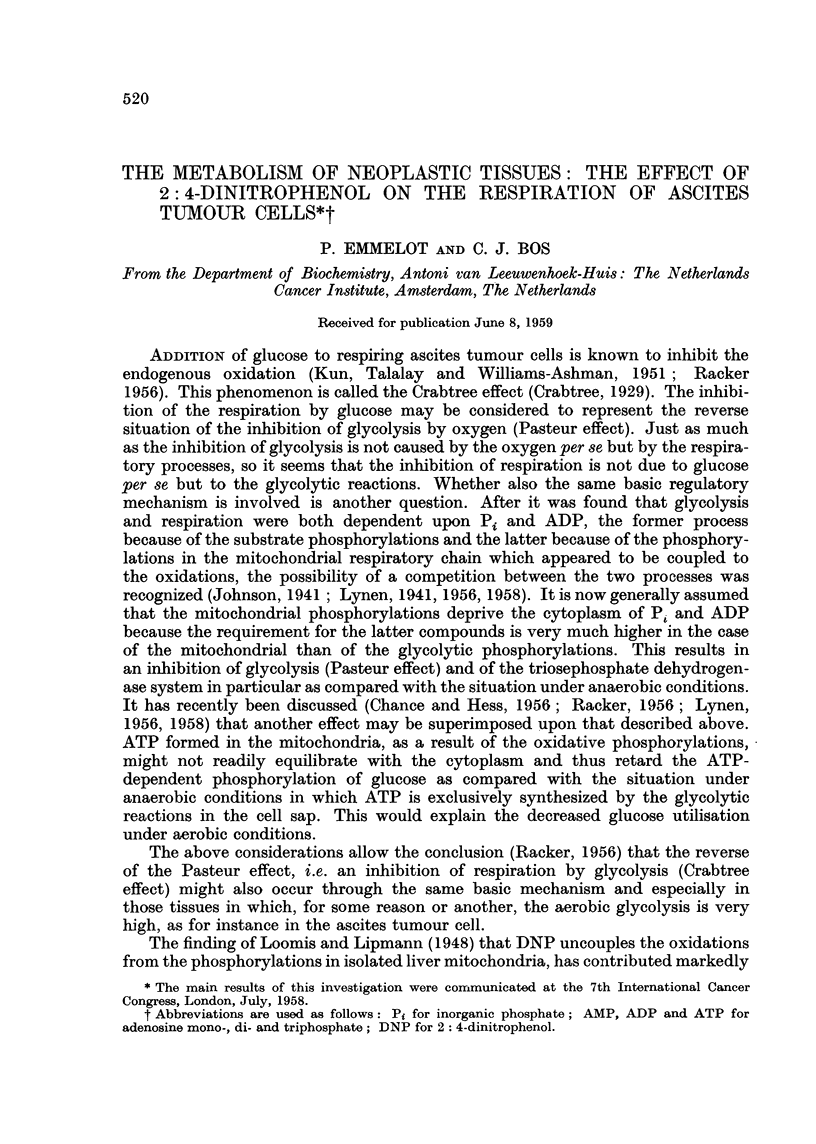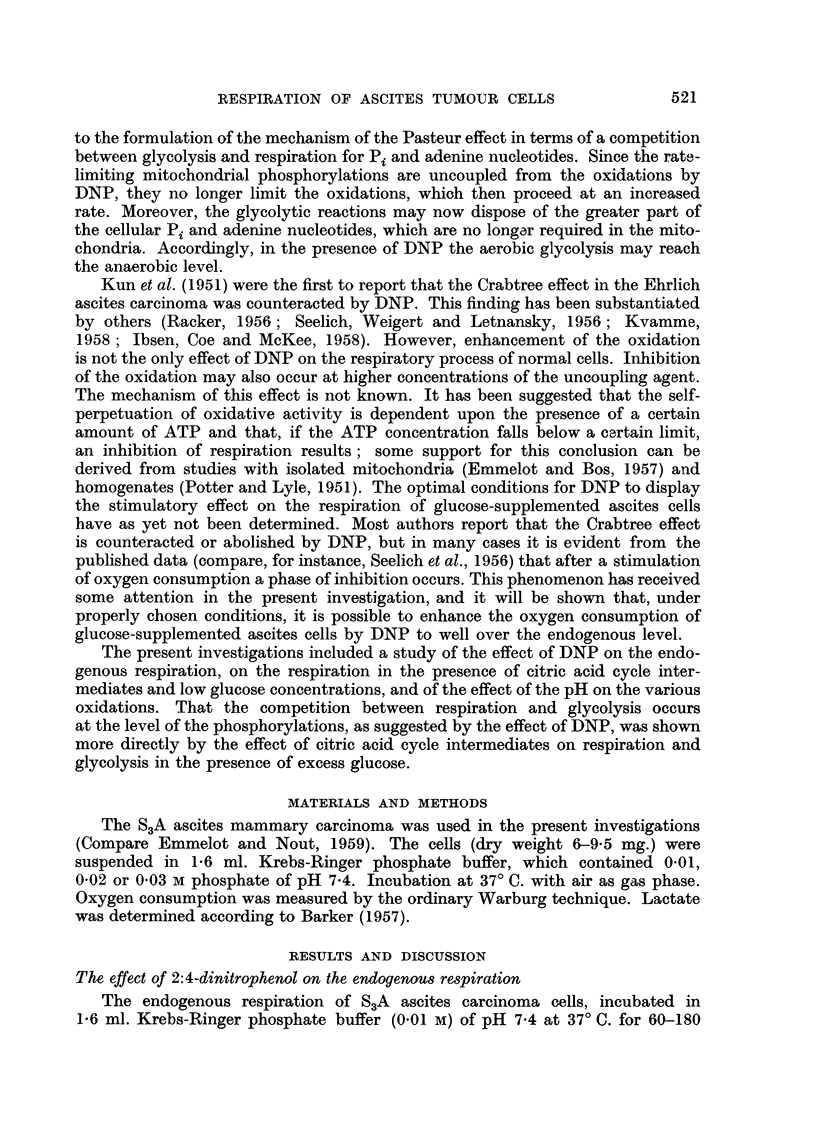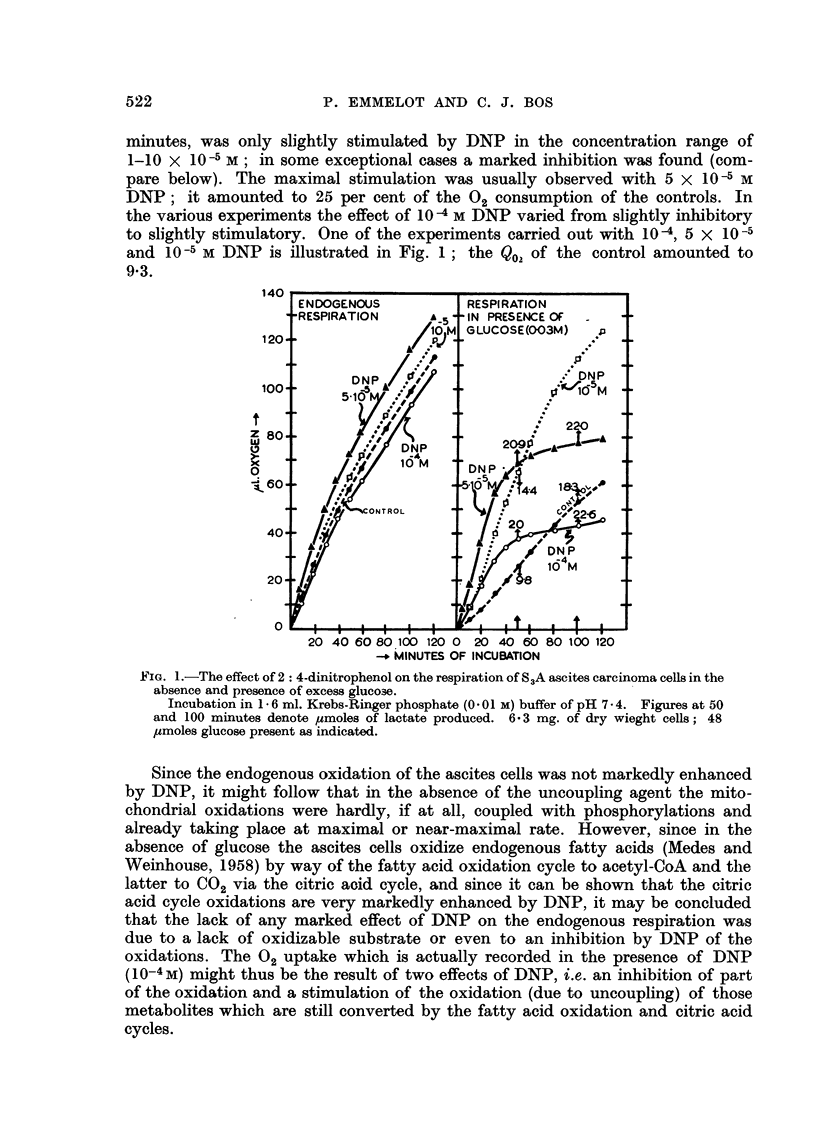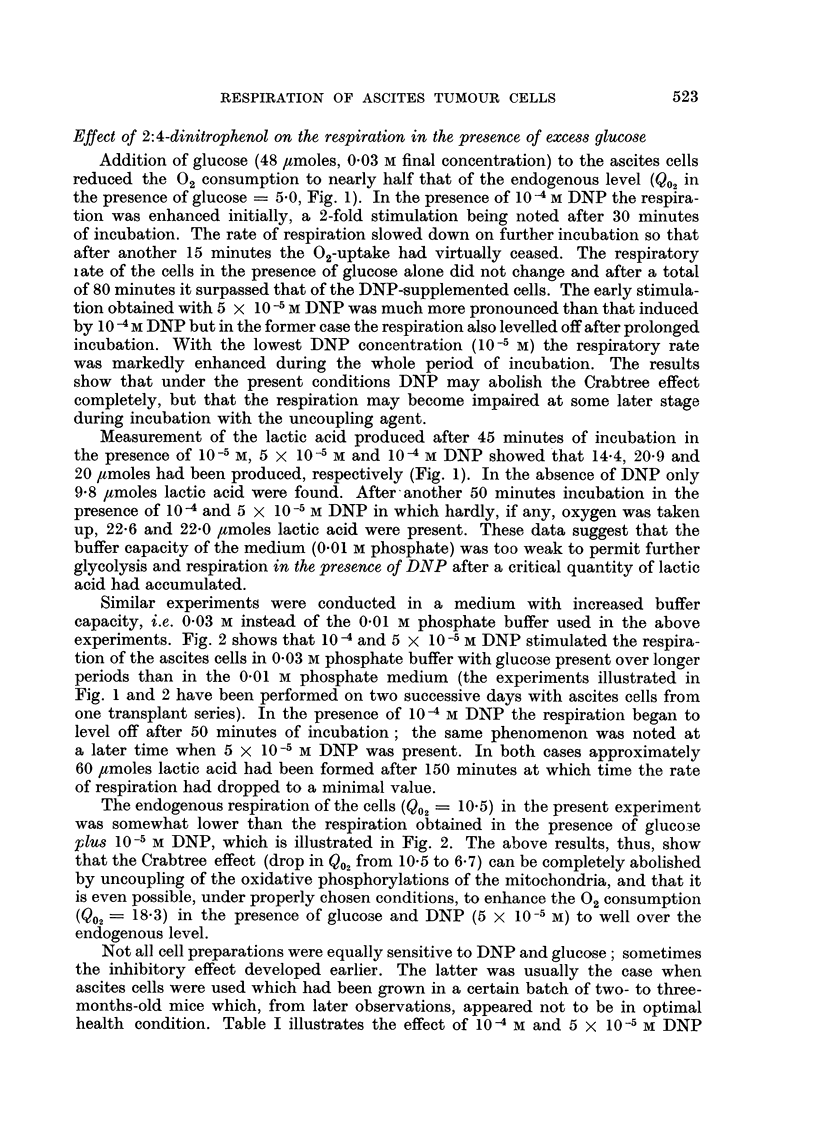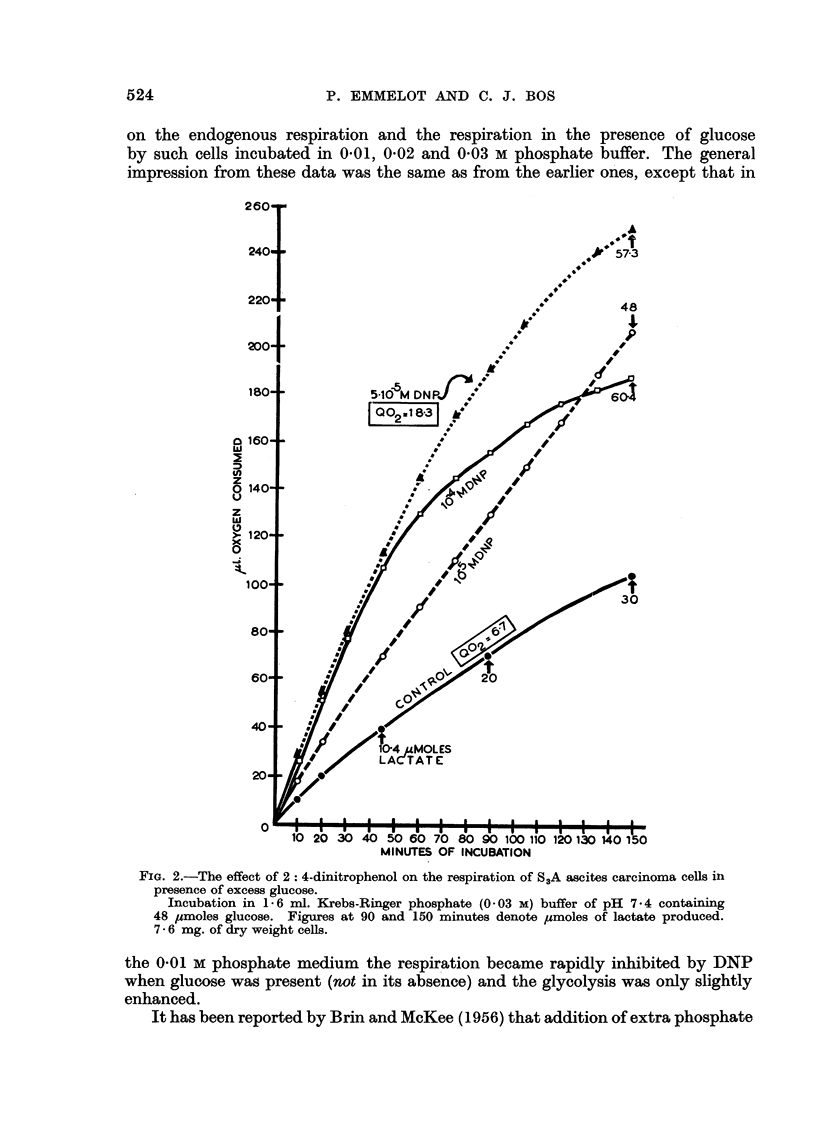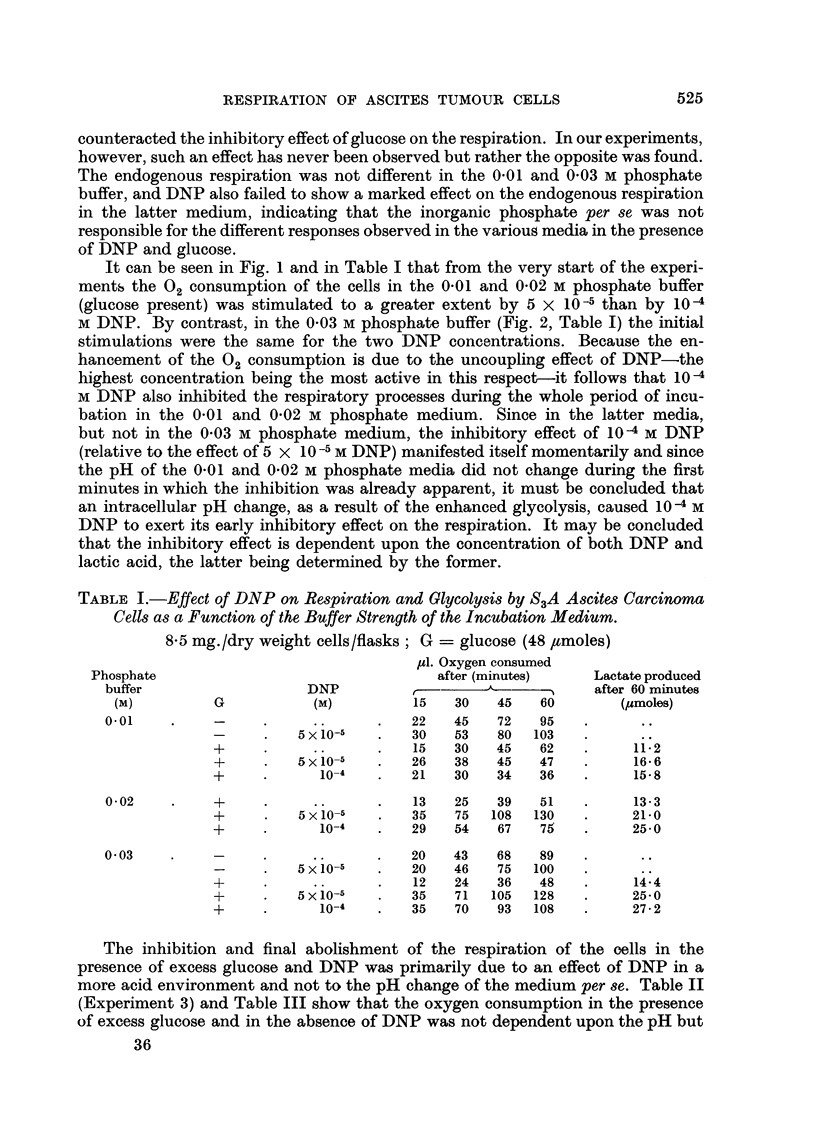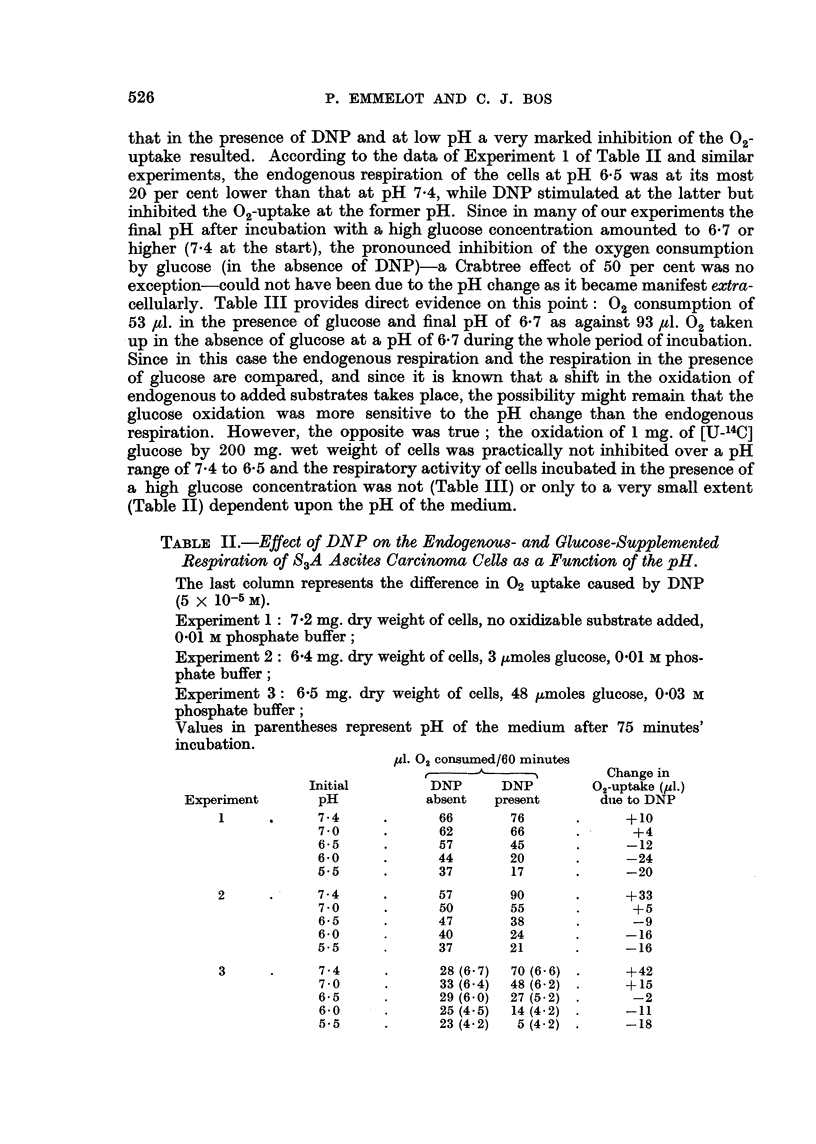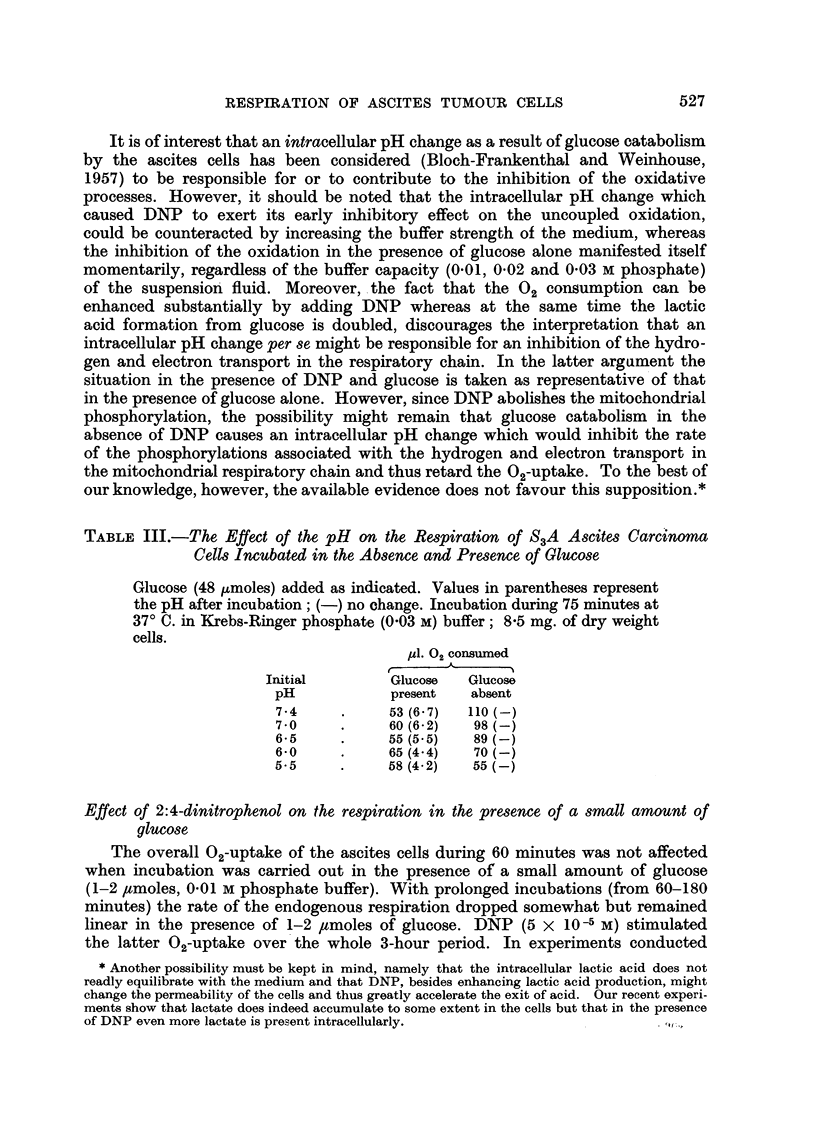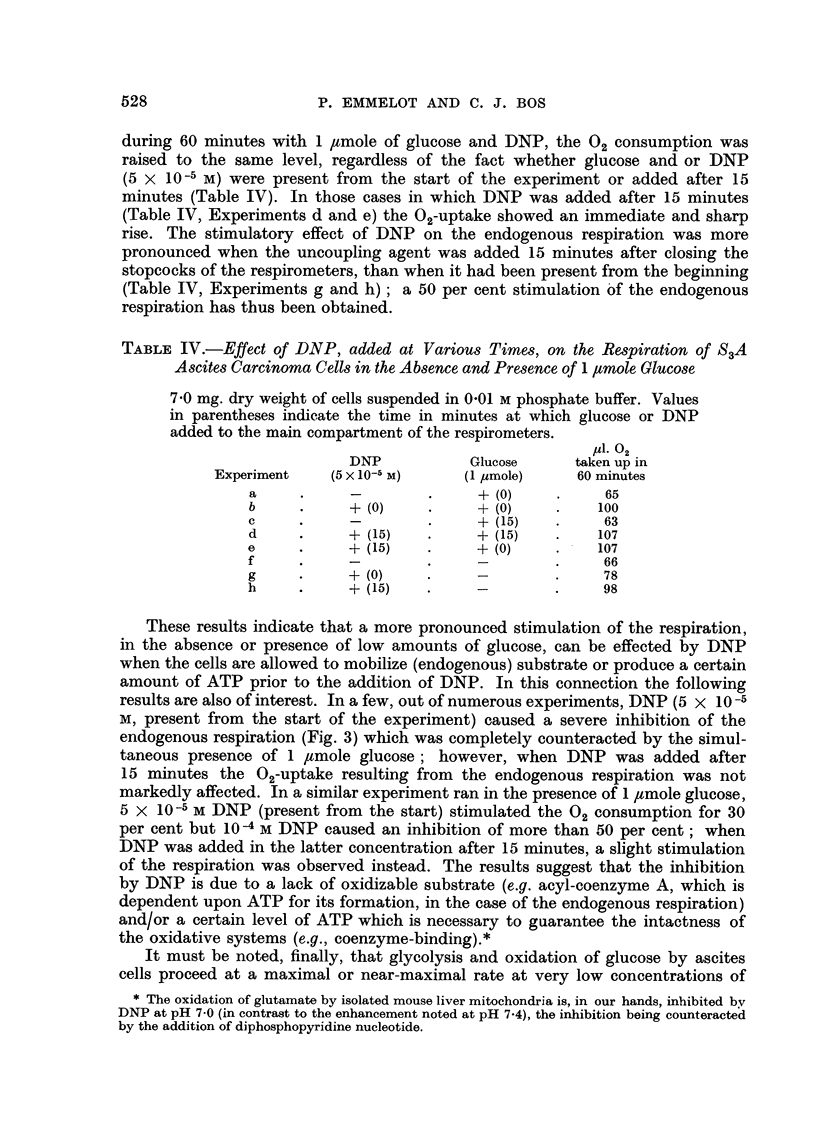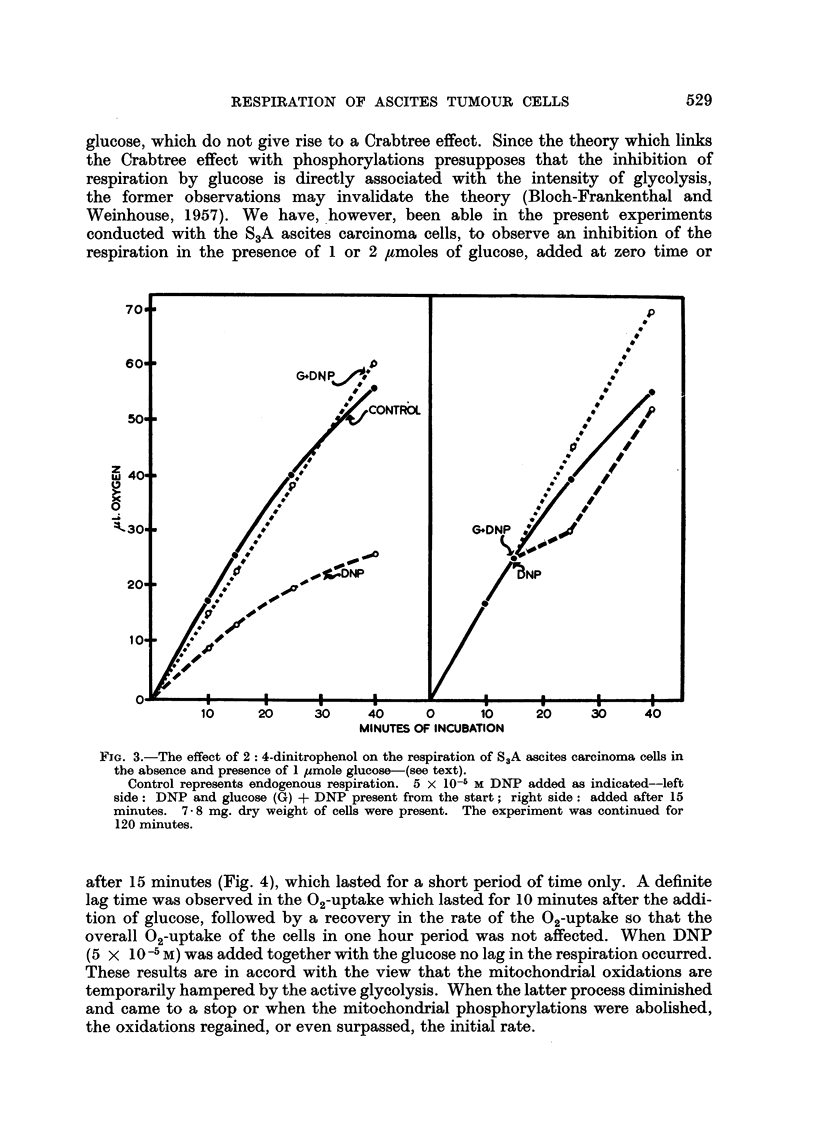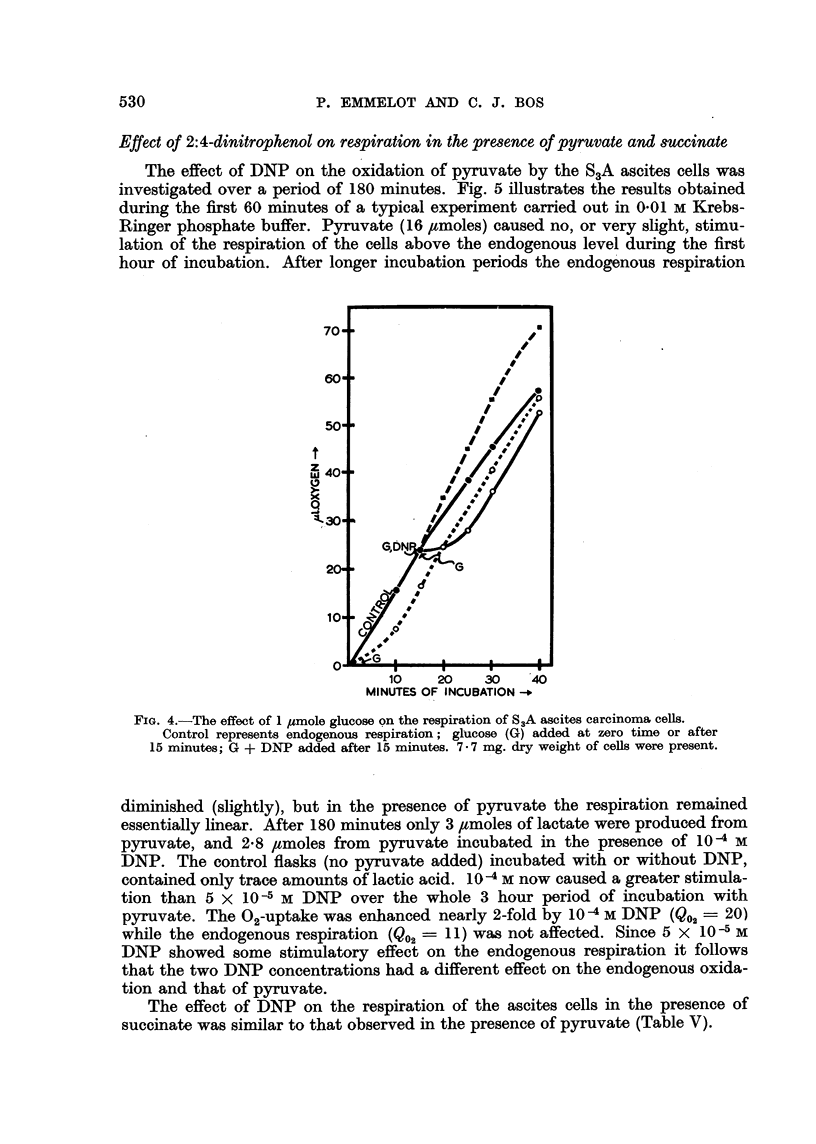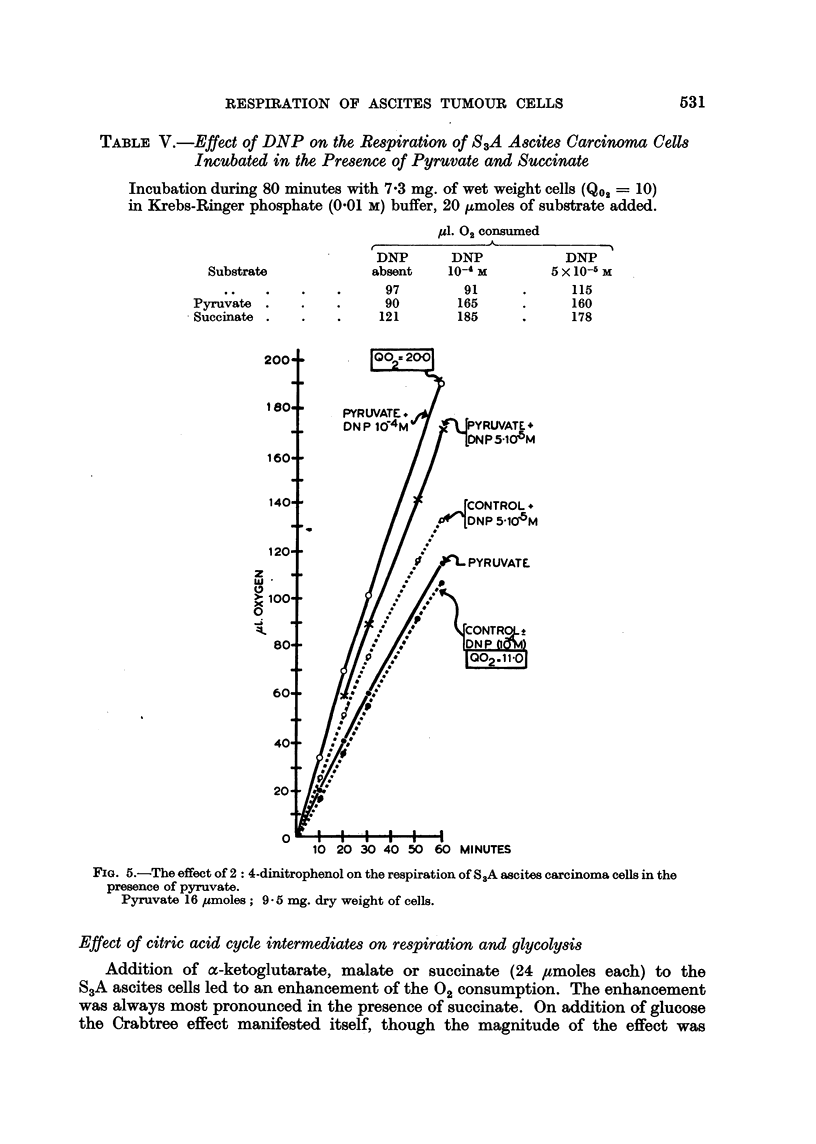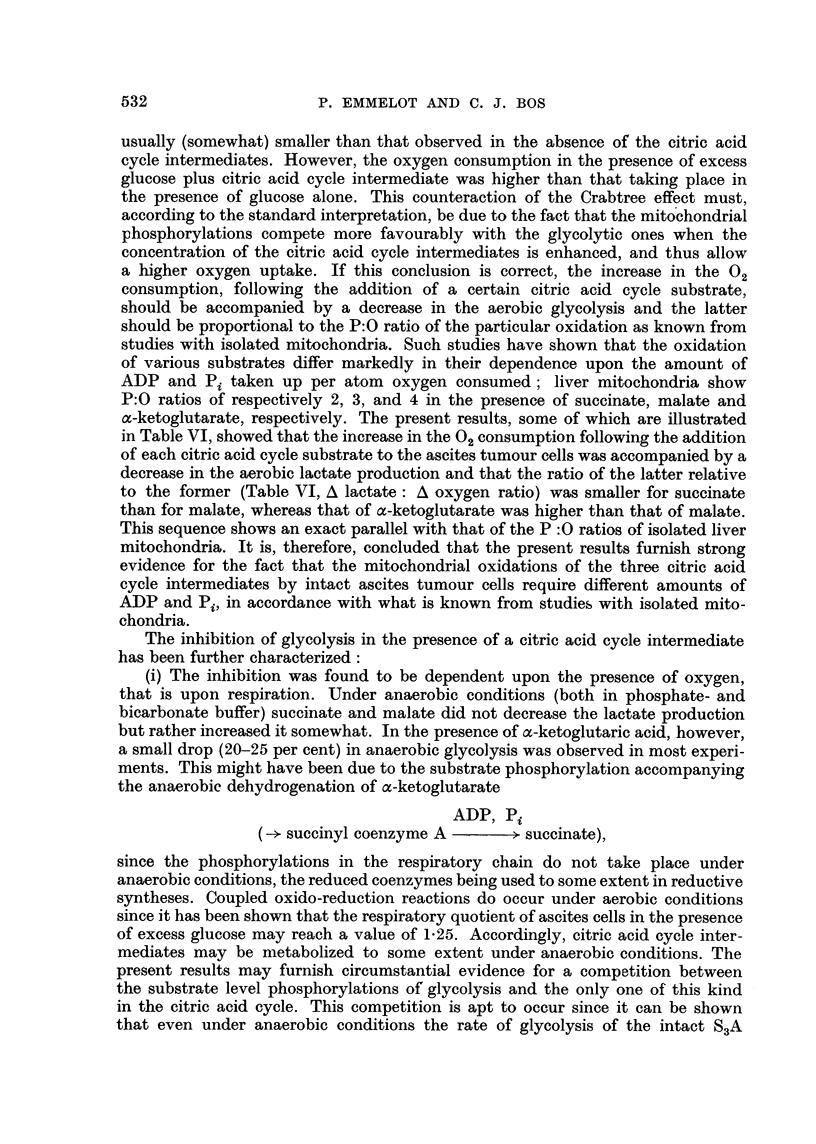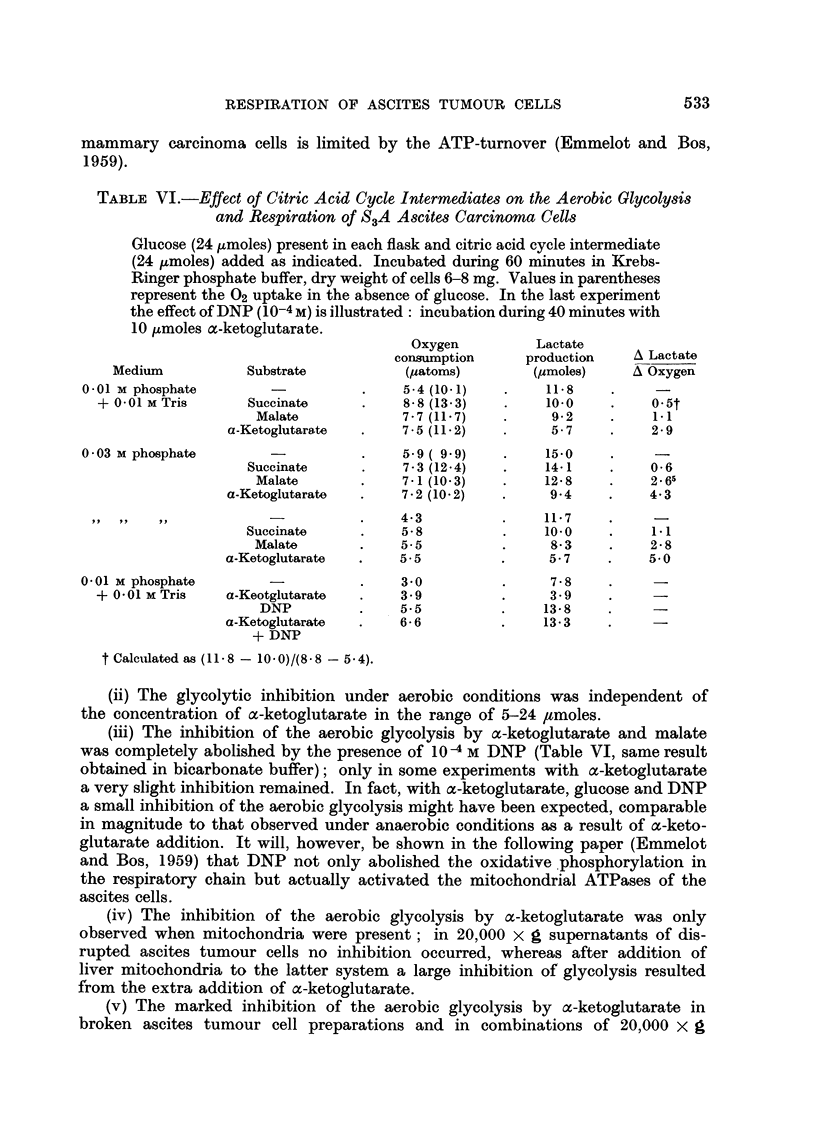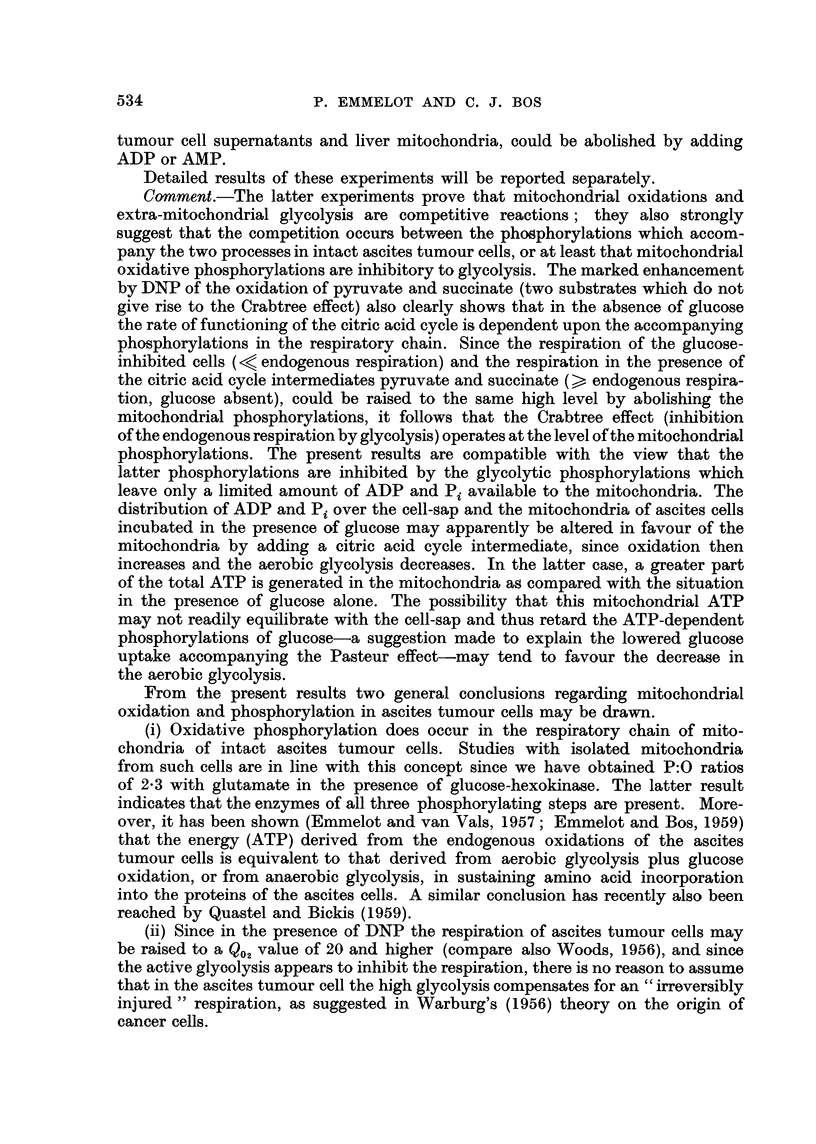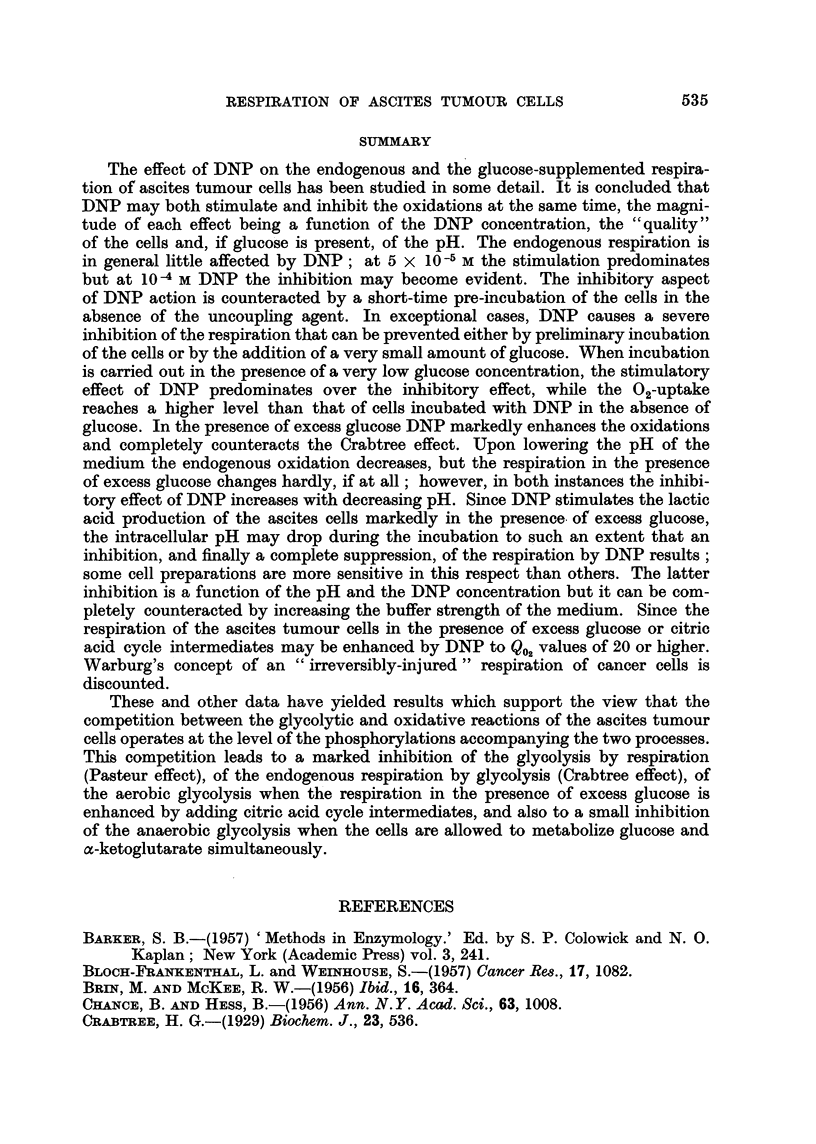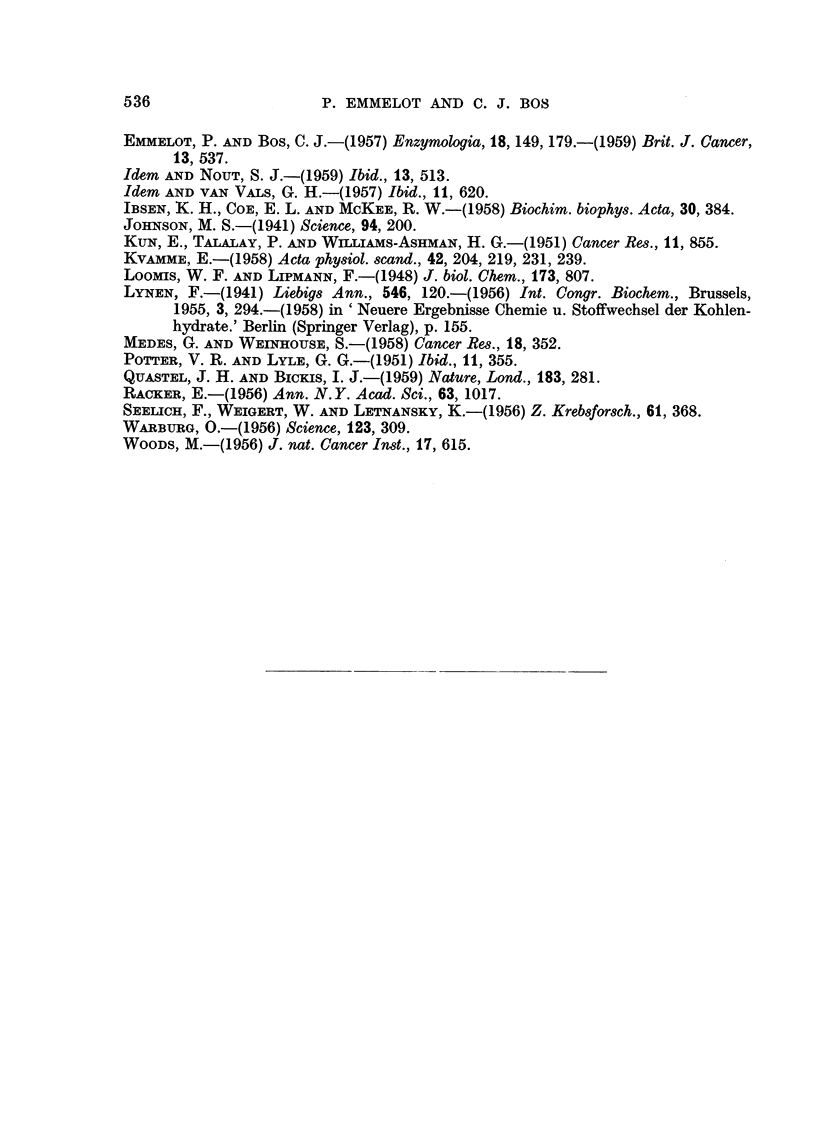# The Metabolism of Neoplastic Tissues: The Effect of 2:4-Dinitrophenol on the Respiration of Ascites Tumour Cells*,†

**DOI:** 10.1038/bjc.1959.58

**Published:** 1959-09

**Authors:** P. Emmelot, C. J. Bos


### THE METABOLISM OF NEOPLASTIC TISSUES: THE EFFECT OF 2:4-DINITROPHENOL ON THE RESPIRATION OF ASCITES TUMOUR CELLS*†

P. EMMELOT AND C. J. BOS

*From the Department of Biochemistry, Antoni van Leeuwenhoek-Huis: The Netherlands Cancer Institute, Amsterdam, The Netherlands*

Received for publication June 8, 1959

ADDITION of glucose to respiring ascites tumour cells is known to inhibit the endogenous oxidation (Kun, Talalay and Williams-Ashman, 1951; Racker 1956). This phenomenon is called the Crabtree effect (Crabtree, 1929). The inhibition of the respiration by glucose may be considered to represent the reverse situation of the inhibition of glycolysis by oxygen (Pasteur effect). Just as much as the inhibition of glycolysis is not caused by the oxygen *per se* but by the respiratory processes, so it seems that the inhibition of respiration is not due to glucose *per se* but to the glycolytic reactions. Whether also the same basic regulatory mechanism is involved is another question. After it was found that glycolysis and respiration were both dependent upon $P_i$ and ADP, the former process because of the substrate phosphorylations and the latter because of the phosphorylations in the mitochondrial respiratory chain which appeared to be coupled to the oxidations, the possibility of a competition between the two processes was recognized (Johnson, 1941; Lynen, 1941, 1956, 1958). It is now generally assumed that the mitochondrial phosphorylations deprive the cytoplasm of $P_i$ and ADP because the requirement for the latter compounds is very much higher in the case of the mitochondrial than of the glycolytic phosphorylations. This results in an inhibition of glycolysis (Pasteur effect) and of the triosephosphate dehydrogenase system in particular as compared with the situation under anaerobic conditions. It has recently been discussed (Chance and Hess, 1956; Racker, 1956; Lynen, 1956, 1958) that another effect may be superimposed upon that described above. ATP formed in the mitochondria, as a result of the oxidative phosphorylations, might not readily equilibrate with the cytoplasm and thus retard the ATP-dependent phosphorylation of glucose as compared with the situation under anaerobic conditions in which ATP is exclusively synthesized by the glycolytic reactions in the cell sap. This would explain the decreased glucose utilisation under aerobic conditions.

The above considerations allow the conclusion (Racker, 1956) that the reverse of the Pasteur effect, *i.e.* an inhibition of respiration by glycolysis (Crabtree effect) might also occur through the same basic mechanism and especially in those tissues in which, for some reason or another, the aerobic glycolysis is very high, as for instance in the ascites tumour cell.

The finding of Loomis and Lipmann (1948) that DNP uncouples the oxidations from the phosphorylations in isolated liver mitochondria, has contributed markedly

* The main results of this investigation were communicated at the 7th International Cancer Congress, London, July, 1958.

† Abbreviations are used as follows: $P_i$ for inorganic phosphate; AMP, ADP and ATP for adenosine mono-, di- and triphosphate; DNP for 2 : 4-dinitrophenol.

to the formulation of the mechanism of the Pasteur effect in terms of a competition between glycolysis and respiration for $P_i$ and adenine nucleotides. Since the rate-limiting mitochondrial phosphorylations are uncoupled from the oxidations by DNP, they no longer limit the oxidations, which then proceed at an increased rate. Moreover, the glycolytic reactions may now dispose of the greater part of the cellular $P_i$ and adenine nucleotides, which are no longer required in the mitochondria. Accordingly, in the presence of DNP the aerobic glycolysis may reach the anaerobic level.

Kun *et al.* (1951) were the first to report that the Crabtree effect in the Ehrlich ascites carcinoma was counteracted by DNP. This finding has been substantiated by others (Racker, 1956 ; Seelich, Weigert and Letnansky, 1956 ; Kvamme, 1958 ; Ibsen, Coe and McKee, 1958). However, enhancement of the oxidation is not the only effect of DNP on the respiratory process of normal cells. Inhibition of the oxidation may also occur at higher concentrations of the uncoupling agent. The mechanism of this effect is not known. It has been suggested that the self-perpetuation of oxidative activity is dependent upon the presence of a certain amount of ATP and that, if the ATP concentration falls below a certain limit, an inhibition of respiration results ; some support for this conclusion can be derived from studies with isolated mitochondria (Emmelot and Bos, 1957) and homogenates (Potter and Lyle, 1951). The optimal conditions for DNP to display the stimulatory effect on the respiration of glucose-supplemented ascites cells have as yet not been determined. Most authors report that the Crabtree effect is counteracted or abolished by DNP, but in many cases it is evident from the published data (compare, for instance, Seelich *et al.*, 1956) that after a stimulation of oxygen consumption a phase of inhibition occurs. This phenomenon has received some attention in the present investigation, and it will be shown that, under properly chosen conditions, it is possible to enhance the oxygen consumption of glucose-supplemented ascites cells by DNP to well over the endogenous level.

The present investigations included a study of the effect of DNP on the endogenous respiration, on the respiration in the presence of citric acid cycle intermediates and low glucose concentrations, and of the effect of the pH on the various oxidations. That the competition between respiration and glycolysis occurs at the level of the phosphorylations, as suggested by the effect of DNP, was shown more directly by the effect of citric acid cycle intermediates on respiration and glycolysis in the presence of excess glucose.

### MATERIALS AND METHODS

The $S_3A$ ascites mammary carcinoma was used in the present investigations (Compare Emmelot and Nout, 1959). The cells (dry weight 6–9·5 mg.) were suspended in 1·6 ml. Krebs-Ringer phosphate buffer, which contained 0·01, 0·02 or 0·03 M phosphate of pH 7·4. Incubation at 37° C. with air as gas phase. Oxygen consumption was measured by the ordinary Warburg technique. Lactate was determined according to Barker (1957).

### RESULTS AND DISCUSSION

### *The effect of 2:4-dinitrophenol on the endogenous respiration*

The endogenous respiration of $S_3A$ ascites carcinoma cells, incubated in 1·6 ml. Krebs-Ringer phosphate buffer (0·01 M) of pH 7·4 at 37° C. for 60–180

minutes, was only slightly stimulated by DNP in the concentration range of 1–10 × $10^{-5}$ M ; in some exceptional cases a marked inhibition was found (compare below). The maximal stimulation was usually observed with 5 × $10^{-5}$ M DNP ; it amounted to 25 per cent of the $O_2$ consumption of the controls. In the various experiments the effect of $10^{-4}$ M DNP varied from slightly inhibitory to slightly stimulatory. One of the experiments carried out with $10^{-4}$, 5 × $10^{-5}$ and $10^{-5}$ M DNP is illustrated in Fig. 1 ; the $Q_{O_2}$ of the control amounted to 9·3.

Fig. 1.—The effect of 2 : 4-dinitrophenol on the respiration of $S_3A$ ascites carcinoma cells in the absence and presence of excess glucose.

Incubation in 1·6 ml. Krebs-Ringer phosphate (0·01 M) buffer of pH 7·4. Figures at 50 and 100 minutes denote μmoles of lactate produced. 6·3 mg. of dry wieght cells ; 48 μmoles glucose present as indicated.

Since the endogenous oxidation of the ascites cells was not markedly enhanced by DNP, it might follow that in the absence of the uncoupling agent the mitochondrial oxidations were hardly, if at all, coupled with phosphorylations and already taking place at maximal or near-maximal rate. However, since in the absence of glucose the ascites cells oxidize endogenous fatty acids (Medes and Weinhouse, 1958) by way of the fatty acid oxidation cycle to acetyl-CoA and the latter to $CO_2$ via the citric acid cycle, and since it can be shown that the citric acid cycle oxidations are very markedly enhanced by DNP, it may be concluded that the lack of any marked effect of DNP on the endogenous respiration was due to a lack of oxidizable substrate or even to an inhibition by DNP of the oxidations. The $O_2$ uptake which is actually recorded in the presence of DNP ($10^{-4}$ M) might thus be the result of two effects of DNP, *i.e.* an inhibition of part of the oxidation and a stimulation of the oxidation (due to uncoupling) of those metabolites which are still converted by the fatty acid oxidation and citric acid cycles.

*Effect of 2:4-dinitrophenol on the respiration in the presence of excess glucose*

Addition of glucose (48 $\mu$moles, 0·03 M final concentration) to the ascites cells reduced the $O_2$ consumption to nearly half that of the endogenous level ($Q_{O_2}$ in the presence of glucose = 5·0, Fig. 1). In the presence of $10^{-4}$ M DNP the respiration was enhanced initially, a 2-fold stimulation being noted after 30 minutes of incubation. The rate of respiration slowed down on further incubation so that after another 15 minutes the $O_2$-uptake had virtually ceased. The respiratory rate of the cells in the presence of glucose alone did not change and after a total of 80 minutes it surpassed that of the DNP-supplemented cells. The early stimulation obtained with $5 \times 10^{-5}$ M DNP was much more pronounced than that induced by $10^{-4}$ M DNP but in the former case the respiration also levelled off after prolonged incubation. With the lowest DNP concentration ($10^{-5}$ M) the respiratory rate was markedly enhanced during the whole period of incubation. The results show that under the present conditions DNP may abolish the Crabtree effect completely, but that the respiration may become impaired at some later stage during incubation with the uncoupling agent.

Measurement of the lactic acid produced after 45 minutes of incubation in the presence of $10^{-5}$ M, $5 \times 10^{-5}$ M and $10^{-4}$ M DNP showed that 14·4, 20·9 and 20 $\mu$moles had been produced, respectively (Fig. 1). In the absence of DNP only 9·8 $\mu$moles lactic acid were found. After another 50 minutes incubation in the presence of $10^{-4}$ and $5 \times 10^{-5}$ M DNP in which hardly, if any, oxygen was taken up, 22·6 and 22·0 $\mu$moles lactic acid were present. These data suggest that the buffer capacity of the medium (0·01 M phosphate) was too weak to permit further glycolysis and respiration *in the presence of DNP* after a critical quantity of lactic acid had accumulated.

Similar experiments were conducted in a medium with increased buffer capacity, *i.e.* 0·03 M instead of the 0·01 M phosphate buffer used in the above experiments. Fig. 2 shows that $10^{-4}$ and $5 \times 10^{-5}$ M DNP stimulated the respiration of the ascites cells in 0·03 M phosphate buffer with glucose present over longer periods than in the 0·01 M phosphate medium (the experiments illustrated in Fig. 1 and 2 have been performed on two successive days with ascites cells from one transplant series). In the presence of $10^{-4}$ M DNP the respiration began to level off after 50 minutes of incubation; the same phenomenon was noted at a later time when $5 \times 10^{-5}$ M DNP was present. In both cases approximately 60 $\mu$moles lactic acid had been formed after 150 minutes at which time the rate of respiration had dropped to a minimal value.

The endogenous respiration of the cells ($Q_{O_2}$ = 10·5) in the present experiment was somewhat lower than the respiration obtained in the presence of glucose *plus* $10^{-5}$ M DNP, which is illustrated in Fig. 2. The above results, thus, show that the Crabtree effect (drop in $Q_{O_2}$ from 10·5 to 6·7) can be completely abolished by uncoupling of the oxidative phosphorylations of the mitochondria, and that it is even possible, under properly chosen conditions, to enhance the $O_2$ consumption ($Q_{O_2}$ = 18·3) in the presence of glucose and DNP ($5 \times 10^{-5}$ M) to well over the endogenous level.

Not all cell preparations were equally sensitive to DNP and glucose; sometimes the inhibitory effect developed earlier. The latter was usually the case when ascites cells were used which had been grown in a certain batch of two- to three-months-old mice which, from later observations, appeared not to be in optimal health condition. Table I illustrates the effect of $10^{-4}$ M and $5 \times 10^{-5}$ M DNP

on the endogenous respiration and the respiration in the presence of glucose by such cells incubated in 0·01, 0·02 and 0·03 M phosphate buffer. The general impression from these data was the same as from the earlier ones, except that in

FIG. 2.—The effect of 2 : 4-dinitrophenol on the respiration of $S_3A$ ascites carcinoma cells in presence of excess glucose.

Incubation in 1·6 ml. Krebs-Ringer phosphate (0·03 M) buffer of pH 7·4 containing 48 µmoles glucose. Figures at 90 and 150 minutes denote µmoles of lactate produced. 7·6 mg. of dry weight cells.

the 0·01 M phosphate medium the respiration became rapidly inhibited by DNP when glucose was present (*not* in its absence) and the glycolysis was only slightly enhanced.

It has been reported by Brin and McKee (1956) that addition of extra phosphate counteracted the inhibitory effect of glucose on the respiration. In our experiments, however, such an effect has never been observed but rather the opposite was found. The endogenous respiration was not different in the 0·01 and 0·03 M phosphate buffer, and DNP also failed to show a marked effect on the endogenous respiration in the latter medium, indicating that the inorganic phosphate *per se* was not responsible for the different responses observed in the various media in the presence of DNP and glucose.

It can be seen in Fig. 1 and in Table I that from the very start of the experiments the $O_2$ consumption of the cells in the 0·01 and 0·02 M phosphate buffer (glucose present) was stimulated to a greater extent by $5 \times 10^{-5}$ than by $10^{-4}$ M DNP. By contrast, in the 0·03 M phosphate buffer (Fig. 2, Table I) the initial stimulations were the same for the two DNP concentrations. Because the enhancement of the $O_2$ consumption is due to the uncoupling effect of DNP—the highest concentration being the most active in this respect—it follows that $10^{-4}$ M DNP also inhibited the respiratory processes during the whole period of incubation in the 0·01 and 0·02 M phosphate medium. Since in the latter media, but not in the 0·03 M phosphate medium, the inhibitory effect of $10^{-4}$ M DNP (relative to the effect of $5 \times 10^{-5}$ M DNP) manifested itself momentarily and since the pH of the 0·01 and 0·02 M phosphate media did not change during the first minutes in which the inhibition was already apparent, it must be concluded that an intracellular pH change, as a result of the enhanced glycolysis, caused $10^{-4}$ M DNP to exert its early inhibitory effect on the respiration. It may be concluded that the inhibitory effect is dependent upon the concentration of both DNP and lactic acid, the latter being determined by the former.

TABLE I.—*Effect of DNP on Respiration and Glycolysis by $S_3A$ Ascites Carcinoma Cells as a Function of the Buffer Strength of the Incubation Medium.*

8·5 mg./dry weight cells/flasks ; G = glucose (48 μmoles)

| Phosphate buffer (M) | G | DNP (M) | μl. Oxygen consumed after (minutes) 15 | 30 | 45 | 60 | Lactate produced after 60 minutes (μmoles) |
|---|---|---|---|---|---|---|---|
| 0·01 | — | .. | 22 | 45 | 72 | 95 | .. |
| | — | $5 \times 10^{-5}$ | 30 | 53 | 80 | 103 | .. |
| | + | .. | 15 | 30 | 45 | 62 | 11·2 |
| | + | $5 \times 10^{-5}$ | 26 | 38 | 45 | 47 | 16·6 |
| | + | $10^{-4}$ | 21 | 30 | 34 | 36 | 15·8 |
| 0·02 | + | .. | 13 | 25 | 39 | 51 | 13·3 |
| | + | $5 \times 10^{-5}$ | 35 | 75 | 108 | 130 | 21·0 |
| | + | $10^{-4}$ | 29 | 54 | 67 | 75 | 25·0 |
| 0·03 | — | .. | 20 | 43 | 68 | 89 | .. |
| | — | $5 \times 10^{-5}$ | 20 | 46 | 75 | 100 | .. |
| | + | .. | 12 | 24 | 36 | 48 | 14·4 |
| | + | $5 \times 10^{-5}$ | 35 | 71 | 105 | 128 | 25·0 |
| | + | $10^{-4}$ | 35 | 70 | 93 | 108 | 27·2 |

The inhibition and final abolishment of the respiration of the cells in the presence of excess glucose and DNP was primarily due to an effect of DNP in a more acid environment and not to the pH change of the medium *per se*. Table II (Experiment 3) and Table III show that the oxygen consumption in the presence of excess glucose and in the absence of DNP was not dependent upon the pH but that in the presence of DNP and at low pH a very marked inhibition of the $O_2$-uptake resulted. According to the data of Experiment 1 of Table II and similar experiments, the endogenous respiration of the cells at pH 6·5 was at its most 20 per cent lower than that at pH 7·4, while DNP stimulated at the latter but inhibited the $O_2$-uptake at the former pH. Since in many of our experiments the final pH after incubation with a high glucose concentration amounted to 6·7 or higher (7·4 at the start), the pronounced inhibition of the oxygen consumption by glucose (in the absence of DNP)—a Crabtree effect of 50 per cent was no exception—could not have been due to the pH change as it became manifest *extra*-cellularly. Table III provides direct evidence on this point: $O_2$ consumption of 53 $\mu$l. in the presence of glucose and final pH of 6·7 as against 93 $\mu$l. $O_2$ taken up in the absence of glucose at a pH of 6·7 during the whole period of incubation. Since in this case the endogenous respiration and the respiration in the presence of glucose are compared, and since it is known that a shift in the oxidation of endogenous to added substrates takes place, the possibility might remain that the glucose oxidation was more sensitive to the pH change than the endogenous respiration. However, the opposite was true; the oxidation of 1 mg. of [U-$^{14}$C] glucose by 200 mg. wet weight of cells was practically not inhibited over a pH range of 7·4 to 6·5 and the respiratory activity of cells incubated in the presence of a high glucose concentration was not (Table III) or only to a very small extent (Table II) dependent upon the pH of the medium.

TABLE II.—*Effect of DNP on the Endogenous- and Glucose-Supplemented Respiration of $S_3A$ Ascites Carcinoma Cells as a Function of the pH.*

The last column represents the difference in $O_2$ uptake caused by DNP ($5 \times 10^{-5}$ M).

Experiment 1: 7·2 mg. dry weight of cells, no oxidizable substrate added, 0·01 M phosphate buffer;

Experiment 2: 6·4 mg. dry weight of cells, 3 $\mu$moles glucose, 0·01 M phosphate buffer;

Experiment 3: 6·5 mg. dry weight of cells, 48 $\mu$moles glucose, 0·03 M phosphate buffer;

Values in parentheses represent pH of the medium after 75 minutes' incubation.

| Experiment | Initial pH | $\mu$l. $O_2$ consumed/60 minutes: DNP absent | DNP present | Change in $O_2$-uptake ($\mu$l.) due to DNP |
|---|---|---|---|---|
| 1 | 7·4 | 66 | 76 | +10 |
| | 7·0 | 62 | 66 | +4 |
| | 6·5 | 57 | 45 | −12 |
| | 6·0 | 44 | 20 | −24 |
| | 5·5 | 37 | 17 | −20 |
| 2 | 7·4 | 57 | 90 | +33 |
| | 7·0 | 50 | 55 | +5 |
| | 6·5 | 47 | 38 | −9 |
| | 6·0 | 40 | 24 | −16 |
| | 5·5 | 37 | 21 | −16 |
| 3 | 7·4 | 28 (6·7) | 70 (6·6) | +42 |
| | 7·0 | 33 (6·4) | 48 (6·2) | +15 |
| | 6·5 | 29 (6·0) | 27 (5·2) | −2 |
| | 6·0 | 25 (4·5) | 14 (4·2) | −11 |
| | 5·5 | 23 (4·2) | 5 (4·2) | −18 |

It is of interest that an *intra*cellular pH change as a result of glucose catabolism by the ascites cells has been considered (Bloch-Frankenthal and Weinhouse, 1957) to be responsible for or to contribute to the inhibition of the oxidative processes. However, it should be noted that the intracellular pH change which caused DNP to exert its early inhibitory effect on the uncoupled oxidation, could be counteracted by increasing the buffer strength of the medium, whereas the inhibition of the oxidation in the presence of glucose alone manifested itself momentarily, regardless of the buffer capacity (0·01, 0·02 and 0·03 M phosphate) of the suspension fluid. Moreover, the fact that the $O_2$ consumption can be enhanced substantially by adding DNP whereas at the same time the lactic acid formation from glucose is doubled, discourages the interpretation that an intracellular pH change *per se* might be responsible for an inhibition of the hydrogen and electron transport in the respiratory chain. In the latter argument the situation in the presence of DNP and glucose is taken as representative of that in the presence of glucose alone. However, since DNP abolishes the mitochondrial phosphorylation, the possibility might remain that glucose catabolism in the absence of DNP causes an intracellular pH change which would inhibit the rate of the phosphorylations associated with the hydrogen and electron transport in the mitochondrial respiratory chain and thus retard the $O_2$-uptake. To the best of our knowledge, however, the available evidence does not favour this supposition.*

TABLE III.—*The Effect of the pH on the Respiration of $S_3A$ Ascites Carcinoma Cells Incubated in the Absence and Presence of Glucose*

Glucose (48 μmoles) added as indicated. Values in parentheses represent the pH after incubation ; (—) no change. Incubation during 75 minutes at 37° C. in Krebs-Ringer phosphate (0·03 M) buffer ; 8·5 mg. of dry weight cells.

| | μl. $O_2$ consumed | |
|---|---|---|
| Initial pH | Glucose present | Glucose absent |
| 7·4 | 53 (6·7) | 110 (—) |
| 7·0 | 60 (6·2) | 98 (—) |
| 6·5 | 55 (5·5) | 89 (—) |
| 6·0 | 65 (4·4) | 70 (—) |
| 5·5 | 58 (4·2) | 55 (—) |

*Effect of 2:4-dinitrophenol on the respiration in the presence of a small amount of glucose*

The overall $O_2$-uptake of the ascites cells during 60 minutes was not affected when incubation was carried out in the presence of a small amount of glucose (1–2 μmoles, 0·01 M phosphate buffer). With prolonged incubations (from 60–180 minutes) the rate of the endogenous respiration dropped somewhat but remained linear in the presence of 1–2 μmoles of glucose. DNP ($5 \times 10^{-5}$ M) stimulated the latter $O_2$-uptake over the whole 3-hour period. In experiments conducted

* Another possibility must be kept in mind, namely that the intracellular lactic acid does not readily equilibrate with the medium and that DNP, besides enhancing lactic acid production, might change the permeability of the cells and thus greatly accelerate the exit of acid. Our recent experiments show that lactate does indeed accumulate to some extent in the cells but that in the presence of DNP even more lactate is present intracellularly.

during 60 minutes with 1 μmole of glucose and DNP, the $O_2$ consumption was raised to the same level, regardless of the fact whether glucose and or DNP ($5 \times 10^{-5}$ M) were present from the start of the experiment or added after 15 minutes (Table IV). In those cases in which DNP was added after 15 minutes (Table IV, Experiments d and e) the $O_2$-uptake showed an immediate and sharp rise. The stimulatory effect of DNP on the endogenous respiration was more pronounced when the uncoupling agent was added 15 minutes after closing the stopcocks of the respirometers, than when it had been present from the beginning (Table IV, Experiments g and h); a 50 per cent stimulation of the endogenous respiration has thus been obtained.

TABLE IV.—*Effect of DNP, added at Various Times, on the Respiration of $S_3A$ Ascites Carcinoma Cells in the Absence and Presence of* 1 *μmole Glucose*

7·0 mg. dry weight of cells suspended in 0·01 M phosphate buffer. Values in parentheses indicate the time in minutes at which glucose or DNP added to the main compartment of the respirometers.

| Experiment | DNP ($5 \times 10^{-5}$ M) | Glucose (1 μmole) | μl. $O_2$ taken up in 60 minutes |
|---|---|---|---|
| a | — | + (0) | 65 |
| b | + (0) | + (0) | 100 |
| c | — | + (15) | 63 |
| d | + (15) | + (15) | 107 |
| e | + (15) | + (0) | 107 |
| f | — | — | 66 |
| g | + (0) | — | 78 |
| h | + (15) | — | 98 |

These results indicate that a more pronounced stimulation of the respiration, in the absence or presence of low amounts of glucose, can be effected by DNP when the cells are allowed to mobilize (endogenous) substrate or produce a certain amount of ATP prior to the addition of DNP. In this connection the following results are also of interest. In a few, out of numerous experiments, DNP ($5 \times 10^{-5}$ M, present from the start of the experiment) caused a severe inhibition of the endogenous respiration (Fig. 3) which was completely counteracted by the simultaneous presence of 1 μmole glucose; however, when DNP was added after 15 minutes the $O_2$-uptake resulting from the endogenous respiration was not markedly affected. In a similar experiment ran in the presence of 1 μmole glucose, $5 \times 10^{-5}$ M DNP (present from the start) stimulated the $O_2$ consumption for 30 per cent but $10^{-4}$ M DNP caused an inhibition of more than 50 per cent; when DNP was added in the latter concentration after 15 minutes, a slight stimulation of the respiration was observed instead. The results suggest that the inhibition by DNP is due to a lack of oxidizable substrate (*e.g.* acyl-coenzyme A, which is dependent upon ATP for its formation, in the case of the endogenous respiration) and/or a certain level of ATP which is necessary to guarantee the intactness of the oxidative systems (*e.g.*, coenzyme-binding).*

It must be noted, finally, that glycolysis and oxidation of glucose by ascites cells proceed at a maximal or near-maximal rate at very low concentrations of

* The oxidation of glutamate by isolated mouse liver mitochondria is, in our hands, inhibited by DNP at pH 7·0 (in contrast to the enhancement noted at pH 7·4), the inhibition being counteracted by the addition of diphosphopyridine nucleotide.

glucose, which do not give rise to a Crabtree effect. Since the theory which links the Crabtree effect with phosphorylations presupposes that the inhibition of respiration by glucose is directly associated with the intensity of glycolysis, the former observations may invalidate the theory (Bloch-Frankenthal and Weinhouse, 1957). We have, however, been able in the present experiments conducted with the $S_3A$ ascites carcinoma cells, to observe an inhibition of the respiration in the presence of 1 or 2 $\mu$moles of glucose, added at zero time or

FIG. 3.—The effect of 2 : 4-dinitrophenol on the respiration of $S_3A$ ascites carcinoma cells in the absence and presence of 1 $\mu$mole glucose—(see text).

Control represents endogenous respiration. $5 \times 10^{-5}$ M DNP added as indicated—left side : DNP and glucose (G) + DNP present from the start ; right side : added after 15 minutes. 7·8 mg. dry weight of cells were present. The experiment was continued for 120 minutes.

after 15 minutes (Fig. 4), which lasted for a short period of time only. A definite lag time was observed in the $O_2$-uptake which lasted for 10 minutes after the addition of glucose, followed by a recovery in the rate of the $O_2$-uptake so that the overall $O_2$-uptake of the cells in one hour period was not affected. When DNP ($5 \times 10^{-5}$ M) was added together with the glucose no lag in the respiration occurred. These results are in accord with the view that the mitochondrial oxidations are temporarily hampered by the active glycolysis. When the latter process diminished and came to a stop or when the mitochondrial phosphorylations were abolished, the oxidations regained, or even surpassed, the initial rate.

*Effect of 2:4-dinitrophenol on respiration in the presence of pyruvate and succinate*

The effect of DNP on the oxidation of pyruvate by the $S_3A$ ascites cells was investigated over a period of 180 minutes. Fig. 5 illustrates the results obtained during the first 60 minutes of a typical experiment carried out in 0·01 M Krebs-Ringer phosphate buffer. Pyruvate (16 $\mu$moles) caused no, or very slight, stimulation of the respiration of the cells above the endogenous level during the first hour of incubation. After longer incubation periods the endogenous respiration

FIG. 4.—The effect of 1 $\mu$mole glucose on the respiration of $S_3A$ ascites carcinoma cells. Control represents endogenous respiration; glucose (G) added at zero time or after 15 minutes; G + DNP added after 15 minutes. 7·7 mg. dry weight of cells were present.

diminished (slightly), but in the presence of pyruvate the respiration remained essentially linear. After 180 minutes only 3 $\mu$moles of lactate were produced from pyruvate, and 2·8 $\mu$moles from pyruvate incubated in the presence of $10^{-4}$ M DNP. The control flasks (no pyruvate added) incubated with or without DNP, contained only trace amounts of lactic acid. $10^{-4}$ M now caused a greater stimulation than $5 \times 10^{-5}$ M DNP over the whole 3 hour period of incubation with pyruvate. The $O_2$-uptake was enhanced nearly 2-fold by $10^{-4}$ M DNP ($Q_{O_2} = 20$) while the endogenous respiration ($Q_{O_2} = 11$) was not affected. Since $5 \times 10^{-5}$ M DNP showed some stimulatory effect on the endogenous respiration it follows that the two DNP concentrations had a different effect on the endogenous oxidation and that of pyruvate.

The effect of DNP on the respiration of the ascites cells in the presence of succinate was similar to that observed in the presence of pyruvate (Table V).

TABLE V.—*Effect of DNP on the Respiration of $S_3A$ Ascites Carcinoma Cells Incubated in the Presence of Pyruvate and Succinate*

Incubation during 80 minutes with 7·3 mg. of wet weight cells ($Q_{O_2} = 10$) in Krebs-Ringer phosphate (0·01 M) buffer, 20 μmoles of substrate added.

| | μl. $O_2$ consumed | | |
|---|---|---|---|
| Substrate | DNP absent | DNP $10^{-4}$ M | DNP $5 \times 10^{-5}$ M |
| .. | 97 | 91 | 115 |
| Pyruvate | 90 | 165 | 160 |
| Succinate | 121 | 185 | 178 |

FIG. 5.—The effect of 2 : 4-dinitrophenol on the respiration of $S_3A$ ascites carcinoma cells in the presence of pyruvate.
Pyruvate 16 μmoles ; 9·5 mg. dry weight of cells.

*Effect of citric acid cycle intermediates on respiration and glycolysis*

Addition of α-ketoglutarate, malate or succinate (24 μmoles each) to the $S_3A$ ascites cells led to an enhancement of the $O_2$ consumption. The enhancement was always most pronounced in the presence of succinate. On addition of glucose the Crabtree effect manifested itself, though the magnitude of the effect was usually (somewhat) smaller than that observed in the absence of the citric acid cycle intermediates. However, the oxygen consumption in the presence of excess glucose plus citric acid cycle intermediate was higher than that taking place in the presence of glucose alone. This counteraction of the Crabtree effect must, according to the standard interpretation, be due to the fact that the mitochondrial phosphorylations compete more favourably with the glycolytic ones when the concentration of the citric acid cycle intermediates is enhanced, and thus allow a higher oxygen uptake. If this conclusion is correct, the increase in the $O_2$ consumption, following the addition of a certain citric acid cycle substrate, should be accompanied by a decrease in the aerobic glycolysis and the latter should be proportional to the P:O ratio of the particular oxidation as known from studies with isolated mitochondria. Such studies have shown that the oxidation of various substrates differ markedly in their dependence upon the amount of ADP and $P_i$ taken up per atom oxygen consumed ; liver mitochondria show P:O ratios of respectively 2, 3, and 4 in the presence of succinate, malate and $\alpha$-ketoglutarate, respectively. The present results, some of which are illustrated in Table VI, showed that the increase in the $O_2$ consumption following the addition of each citric acid cycle substrate to the ascites tumour cells was accompanied by a decrease in the aerobic lactate production and that the ratio of the latter relative to the former (Table VI, $\Delta$ lactate : $\Delta$ oxygen ratio) was smaller for succinate than for malate, whereas that of $\alpha$-ketoglutarate was higher than that of malate. This sequence shows an exact parallel with that of the P :O ratios of isolated liver mitochondria. It is, therefore, concluded that the present results furnish strong evidence for the fact that the mitochondrial oxidations of the three citric acid cycle intermediates by intact ascites tumour cells require different amounts of ADP and $P_i$, in accordance with what is known from studies with isolated mitochondria.

The inhibition of glycolysis in the presence of a citric acid cycle intermediate has been further characterized :

(i) The inhibition was found to be dependent upon the presence of oxygen, that is upon respiration. Under anaerobic conditions (both in phosphate- and bicarbonate buffer) succinate and malate did not decrease the lactate production but rather increased it somewhat. In the presence of $\alpha$-ketoglutaric acid, however, a small drop (20–25 per cent) in anaerobic glycolysis was observed in most experiments. This might have been due to the substrate phosphorylation accompanying the anaerobic dehydrogenation of $\alpha$-ketoglutarate

$$(\rightarrow \text{succinyl coenzyme A} \xrightarrow{\text{ADP, } P_i} \text{succinate}),$$

since the phosphorylations in the respiratory chain do not take place under anaerobic conditions, the reduced coenzymes being used to some extent in reductive syntheses. Coupled oxido-reduction reactions do occur under aerobic conditions since it has been shown that the respiratory quotient of ascites cells in the presence of excess glucose may reach a value of 1·25. Accordingly, citric acid cycle intermediates may be metabolized to some extent under anaerobic conditions. The present results may furnish circumstantial evidence for a competition between the substrate level phosphorylations of glycolysis and the only one of this kind in the citric acid cycle. This competition is apt to occur since it can be shown that even under anaerobic conditions the rate of glycolysis of the intact $S_3A$

mammary carcinoma cells is limited by the ATP-turnover (Emmelot and Bos, 1959).

TABLE VI.—*Effect of Citric Acid Cycle Intermediates on the Aerobic Glycolysis and Respiration of $S_3A$ Ascites Carcinoma Cells*

Glucose (24 μmoles) present in each flask and citric acid cycle intermediate (24 μmoles) added as indicated. Incubated during 60 minutes in Krebs-Ringer phosphate buffer, dry weight of cells 6–8 mg. Values in parentheses represent the $O_2$ uptake in the absence of glucose. In the last experiment the effect of DNP ($10^{-4}$ M) is illustrated : incubation during 40 minutes with 10 μmoles α-ketoglutarate.

| Medium | Substrate | Oxygen consumption (μatoms) | Lactate production (μmoles) | Δ Lactate / Δ Oxygen |
|---|---|---|---|---|
| 0·01 M phosphate | — | 5·4 (10·1) | 11·8 | — |
| + 0·01 M Tris | Succinate | 8·8 (13·3) | 10·0 | 0·5† |
| | Malate | 7·7 (11·7) | 9·2 | 1·1 |
| | α-Ketoglutarate | 7·5 (11·2) | 5·7 | 2·9 |
| 0·03 M phosphate | — | 5·9 ( 9·9) | 15·0 | — |
| | Succinate | 7·3 (12·4) | 14·1 | 0·6 |
| | Malate | 7·1 (10·3) | 12·8 | 2·6⁵ |
| | α-Ketoglutarate | 7·2 (10·2) | 9·4 | 4·3 |
| ,, ,, ,, | — | 4·3 | 11·7 | — |
| | Succinate | 5·8 | 10·0 | 1·1 |
| | Malate | 5·5 | 8·3 | 2·8 |
| | α-Ketoglutarate | 5·5 | 5·7 | 5·0 |
| 0·01 M phosphate | — | 3·0 | 7·8 | — |
| + 0·01 M Tris | α-Keotglutarate | 3·9 | 3·9 | — |
| | DNP | 5·5 | 13·8 | — |
| | α-Ketoglutarate + DNP | 6·6 | 13·3 | — |

† Calculated as (11·8 − 10·0)/(8·8 − 5·4).

(ii) The glycolytic inhibition under aerobic conditions was independent of the concentration of α-ketoglutarate in the range of 5–24 μmoles.

(iii) The inhibition of the aerobic glycolysis by α-ketoglutarate and malate was completely abolished by the presence of $10^{-4}$ M DNP (Table VI, same result obtained in bicarbonate buffer); only in some experiments with α-ketoglutarate a very slight inhibition remained. In fact, with α-ketoglutarate, glucose and DNP a small inhibition of the aerobic glycolysis might have been expected, comparable in magnitude to that observed under anaerobic conditions as a result of α-ketoglutarate addition. It will, however, be shown in the following paper (Emmelot and Bos, 1959) that DNP not only abolished the oxidative phosphorylation in the respiratory chain but actually activated the mitochondrial ATPases of the ascites cells.

(iv) The inhibition of the aerobic glycolysis by α-ketoglutarate was only observed when mitochondria were present ; in 20,000 × g supernatants of disrupted ascites tumour cells no inhibition occurred, whereas after addition of liver mitochondria to the latter system a large inhibition of glycolysis resulted from the extra addition of α-ketoglutarate.

(v) The marked inhibition of the aerobic glycolysis by α-ketoglutarate in broken ascites tumour cell preparations and in combinations of 20,000 × g tumour cell supernatants and liver mitochondria, could be abolished by adding ADP or AMP.

Detailed results of these experiments will be reported separately.

*Comment.*—The latter experiments prove that mitochondrial oxidations and extra-mitochondrial glycolysis are competitive reactions; they also strongly suggest that the competition occurs between the phosphorylations which accompany the two processes in intact ascites tumour cells, or at least that mitochondrial oxidative phosphorylations are inhibitory to glycolysis. The marked enhancement by DNP of the oxidation of pyruvate and succinate (two substrates which do not give rise to the Crabtree effect) also clearly shows that in the absence of glucose the rate of functioning of the citric acid cycle is dependent upon the accompanying phosphorylations in the respiratory chain. Since the respiration of the glucose-inhibited cells ($\ll$ endogenous respiration) and the respiration in the presence of the citric acid cycle intermediates pyruvate and succinate ($\geqslant$ endogenous respiration, glucose absent), could be raised to the same high level by abolishing the mitochondrial phosphorylations, it follows that the Crabtree effect (inhibition of the endogenous respiration by glycolysis) operates at the level of the mitochondrial phosphorylations. The present results are compatible with the view that the latter phosphorylations are inhibited by the glycolytic phosphorylations which leave only a limited amount of ADP and $P_i$ available to the mitochondria. The distribution of ADP and $P_i$ over the cell-sap and the mitochondria of ascites cells incubated in the presence of glucose may apparently be altered in favour of the mitochondria by adding a citric acid cycle intermediate, since oxidation then increases and the aerobic glycolysis decreases. In the latter case, a greater part of the total ATP is generated in the mitochondria as compared with the situation in the presence of glucose alone. The possibility that this mitochondrial ATP may not readily equilibrate with the cell-sap and thus retard the ATP-dependent phosphorylations of glucose—a suggestion made to explain the lowered glucose uptake accompanying the Pasteur effect—may tend to favour the decrease in the aerobic glycolysis.

From the present results two general conclusions regarding mitochondrial oxidation and phosphorylation in ascites tumour cells may be drawn.

(i) Oxidative phosphorylation does occur in the respiratory chain of mitochondria of intact ascites tumour cells. Studies with isolated mitochondria from such cells are in line with this concept since we have obtained P:O ratios of 2·3 with glutamate in the presence of glucose-hexokinase. The latter result indicates that the enzymes of all three phosphorylating steps are present. Moreover, it has been shown (Emmelot and van Vals, 1957; Emmelot and Bos, 1959) that the energy (ATP) derived from the endogenous oxidations of the ascites tumour cells is equivalent to that derived from aerobic glycolysis plus glucose oxidation, or from anaerobic glycolysis, in sustaining amino acid incorporation into the proteins of the ascites cells. A similar conclusion has recently also been reached by Quastel and Bickis (1959).

(ii) Since in the presence of DNP the respiration of ascites tumour cells may be raised to a $Q_{O_2}$ value of 20 and higher (compare also Woods, 1956), and since the active glycolysis appears to inhibit the respiration, there is no reason to assume that in the ascites tumour cell the high glycolysis compensates for an "irreversibly injured" respiration, as suggested in Warburg's (1956) theory on the origin of cancer cells.

### SUMMARY

The effect of DNP on the endogenous and the glucose-supplemented respiration of ascites tumour cells has been studied in some detail. It is concluded that DNP may both stimulate and inhibit the oxidations at the same time, the magnitude of each effect being a function of the DNP concentration, the "quality" of the cells and, if glucose is present, of the pH. The endogenous respiration is in general little affected by DNP ; at $5 \times 10^{-5}$ M the stimulation predominates but at $10^{-4}$ M DNP the inhibition may become evident. The inhibitory aspect of DNP action is counteracted by a short-time pre-incubation of the cells in the absence of the uncoupling agent. In exceptional cases, DNP causes a severe inhibition of the respiration that can be prevented either by preliminary incubation of the cells or by the addition of a very small amount of glucose. When incubation is carried out in the presence of a very low glucose concentration, the stimulatory effect of DNP predominates over the inhibitory effect, while the $O_2$-uptake reaches a higher level than that of cells incubated with DNP in the absence of glucose. In the presence of excess glucose DNP markedly enhances the oxidations and completely counteracts the Crabtree effect. Upon lowering the pH of the medium the endogenous oxidation decreases, but the respiration in the presence of excess glucose changes hardly, if at all ; however, in both instances the inhibitory effect of DNP increases with decreasing pH. Since DNP stimulates the lactic acid production of the ascites cells markedly in the presence of excess glucose, the intracellular pH may drop during the incubation to such an extent that an inhibition, and finally a complete suppression, of the respiration by DNP results ; some cell preparations are more sensitive in this respect than others. The latter inhibition is a function of the pH and the DNP concentration but it can be completely counteracted by increasing the buffer strength of the medium. Since the respiration of the ascites tumour cells in the presence of excess glucose or citric acid cycle intermediates may be enhanced by DNP to $Q_{O_2}$ values of 20 or higher. Warburg's concept of an "irreversibly-injured" respiration of cancer cells is discounted.

These and other data have yielded results which support the view that the competition between the glycolytic and oxidative reactions of the ascites tumour cells operates at the level of the phosphorylations accompanying the two processes. This competition leads to a marked inhibition of the glycolysis by respiration (Pasteur effect), of the endogenous respiration by glycolysis (Crabtree effect), of the aerobic glycolysis when the respiration in the presence of excess glucose is enhanced by adding citric acid cycle intermediates, and also to a small inhibition of the anaerobic glycolysis when the cells are allowed to metabolize glucose and α-ketoglutarate simultaneously.

### REFERENCES

Barker, S. B.—(1957) 'Methods in Enzymology.' Ed. by S. P. Colowick and N. O. Kaplan ; New York (Academic Press) vol. 3, 241.

Bloch-Frankenthal, L. and Weinhouse, S.—(1957) *Cancer Res.*, **17**, 1082.

Brin, M. and McKee, R. W.—(1956) *Ibid.*, **16**, 364.

Chance, B. and Hess, B.—(1956) *Ann. N.Y. Acad. Sci.*, **63**, 1008.

Crabtree, H. G.—(1929) *Biochem. J.*, **23**, 536.

EMMELOT, P. AND BOS, C. J.—(1957) *Enzymologia*, **18**, 149, 179.—(1959) *Brit. J. Cancer*, **13**, 537.
*Idem* AND NOUT, S. J.—(1959) *Ibid.*, **13**, 513.
*Idem* AND VAN VALS, G. H.—(1957) *Ibid.*, **11**, 620.
IBSEN, K. H., COE, E. L. AND MCKEE, R. W.—(1958) *Biochim. biophys. Acta*, **30**, 384.
JOHNSON, M. S.—(1941) *Science*, **94**, 200.
KUN, E., TALALAY, P. AND WILLIAMS-ASHMAN, H. G.—(1951) *Cancer Res.*, **11**, 855.
KVAMME, E.—(1958) *Acta physiol. scand.*, **42**, 204, 219, 231, 239.
LOOMIS, W. F. AND LIPMANN, F.—(1948) *J. biol. Chem.*, **173**, 807.
LYNEN, F.—(1941) *Liebigs Ann.*, **546**, 120.—(1956) *Int. Congr. Biochem.*, Brussels, 1955, **3**, 294.—(1958) in ' Neuere Ergebnisse Chemie u. Stoffwechsel der Kohlenhydrate.' Berlin (Springer Verlag), p. 155.
MEDES, G. AND WEINHOUSE, S.—(1958) *Cancer Res.*, **18**, 352.
POTTER, V. R. AND LYLE, G. G.—(1951) *Ibid.*, **11**, 355.
QUASTEL, J. H. AND BICKIS, I. J.—(1959) *Nature, Lond.*, **183**, 281.
RACKER, E.—(1956) *Ann. N.Y. Acad. Sci.*, **63**, 1017.
SEELICH, F., WEIGERT, W. AND LETNANSKY, K.—(1956) *Z. Krebsforsch.*, **61**, 368.
WARBURG, O.—(1956) *Science*, **123**, 309.
WOODS, M.—(1956) *J. nat. Cancer Inst.*, **17**, 615.